# Monitoring of new psychoactive substances in France: update of addictovigilance data

**DOI:** 10.1093/eurpub/ckag106

**Published:** 2026-06-16

**Authors:** Arnaud Autellet, Clémence Lacroix, Anne Batisse, Joëlle Micallef, Nathalie Fouilhe, Reynald Le Boisselier, Hélène Peyrière, Céline Eiden, French Addictovigilance Network, Amélie Daveluy, Célian Bertin, Johan Thiery, Caroline Victorri-Vigneau, Giulia Panfili, Alexandra Boucher, Louise Carton, Emilie Jouanjus

**Affiliations:** Centre d‘addictovigilance Occitanie-Est, CHU Montpellier, Montpellier, France; Centre d‘addictovigilance Provence-Alpes-Côte d’Azur et Corse, AP-HM, Marseille, France; Centre d‘addictovigilance Ile de France et Centre, APHP, Paris, France; Centre d‘addictovigilance Provence-Alpes-Côte d’Azur et Corse, AP-HM, Marseille, France; Centre d‘addictovigilance Rhône-Alpes Est, CHU Grenoble Alpes, Grenoble, France; Centre d‘addictovigilance Bretagne Normandie, CHU Caen, Caen, France; Centre d‘addictovigilance Occitanie-Est, CHU Montpellier, Montpellier, France; Centre d‘addictovigilance Occitanie-Est, CHU Montpellier, Montpellier, France

## Abstract

New psychoactive substances (NPS) represent a growing public health challenge in Europe. Their rapid emergence, chemical diversity and unpredictable toxicity complicate prevention, clinical management and regulatory responses. This study provides an updated overview of NPS-related harms in France using national addictovigilance data. Data were extracted from three complementary surveillance systems: (i) the NotS program of spontaneous reports of NPS-related adverse events (2009–24), (ii) the OPPIDUM survey conducted among users attending addiction care centers (2016–24), and (iii) the DRAMES registry of toxicologically confirmed drug-related deaths (2012–23). Substances were classified by chemical family and clinical effects were coded using MedDRA terminology. In addition to descriptive analyses, regression models were used to assess temporal trends. A total of 3468 NotS involving at least one NPS were recorded, increasing from 5 cases in 2009 to 726 in 2024. Synthetic cathinones and synthetic cannabinoids accounted for nearly 90% of notifications. Trend analyses confirmed a significant increase in NPS notifications and reported use over time. In OPPIDUM, the proportion of NPS users increased from 0.3% in 2016 to 1.7% in 2024. In DRAMES, 136 NPS-related deaths were identified between 2012 and 2023, with no significant overall increase in mortality during the study period. Synthetic cathinones and synthetic cannabinoids dominate the French NPS landscape. The integration of multiple addictovigilance data sources provides a robust framework for early signal detection and supports public health decision-making.

## Introduction

New psychoactive substances (NPS) have emerged over the past two decades as a major public health challenge worldwide [1]. These compounds are designed to mimic the effects of controlled substances such as cannabis, stimulants, hallucinogens, or opioids. Their rapid chemical modifications allow them to circumvent drug legislation and may limit their detection by standard toxicological screening [2]. In France, NPS use has been associated with acute toxicological emergencies, psychiatric and somatic adverse events, and, in some cases, fatal outcomes [3]. The rapid evolution, unpredictable pharmacological potency, and frequent adulteration of traditional psychoactive substances (PS) complicate both clinical management and regulatory control [4].

In France, monitoring of PS relies on two complementary institutions: the French Medicines Agency (ANSM), which coordinates the French Addictovigilance Network (FAN) and the French Monitoring Centre for Drugs and Drug Addiction (OFDT) [5–7].

Both institutions contribute to the European Early Warning System (EWS) coordinated by the European Union Drugs Agency (EUDA), enabling rapid sharing of information on emerging substances across Europe [1].

Since the early 2000s ∼450 distinct NPS have been identified in France; with 17 newly reported in 2023 alone [7]. In 2017, ∼1.3% of adults (18–64 years) reported lifetime use of synthetic cannabinoid (SC), with higher rates among adults under 35 (up to 3.5%) [8]. Among adolescents, 3.8% of 17-year-olds reported using a product mimicking PS; of whom 11.8% specified it was a SC [9].

Between 2009 and 2017, the FAN received ∼800 reports of NPS-related abuse or adverse events, including 71 fatalities (9%). Synthetic cathinones accounted for the largest share of reported deaths (43%), followed by dissociatives (22%) and designer medicines such as synthetic opioids and designer benzodiazepines (17%) [10].

This study aims to provide an updated overview of addictovigilance data on NPS in France. In addition to cumulative analyses since 2009, we performed a detailed assessment of 2024 data across substance classes to describe current trends in availability, use, and associated adverse events using national monitoring systems (NotS, OPPIDUM, and DRAMES).

## Methods

This study is based on the analysis of national addictovigilance data collected in France through three complementary surveillance programs [6].

### National addictovigilance database (ANPV)

The National addictovigilance database (ANPV) compiles notifications (NotS) of adverse events associated with PS, reported regional addictovigilance centers [11, 12].

Under the French Public Health Code, notification of serious cases of abuse, dependence or harmful use of PS is mandatory for physicians, dentists, midwives, and pharmacists [13].

The FAN is a public health surveillance system dedicated to monitoring cases of abuse and dependence related to psychoactive substances (prescription or illicit), excluding alcohol and tobacco. Reports can be made by healthcare professionals working in healthcare facilities (hospital departments, toxicology laboratories, and poison control centers) or by users through dedicated reporting channels (telephone or online forms). Reports can be anonymous, provided that the clinical information necessary for assessment is available.

The identification of NPS relies on information provided by the reporting person (e.g. patient statement, product name or packaging, or information obtained at the time of purchase) and, if available, on toxicological analyses. Since toxicological confirmation is not always required, the level of certainty can vary from case to case.

All NotS involving NPS between 2009 and 2024 were extracted. Each notification corresponds to a clinical case. Multiple notifications may occur for the same individual if separate clinical events are reported. Upon receipt, cases are first reviewed at the regional level to ensure they are assigned to the appropriate addictovigilance center. A systematic de-duplication procedure is performed prior to entry into the national database, based on available identifiers such as age, sex and clinical characteristics, to avoid duplicate recording of the same event. Analyses were conducted at the case level after this de-duplication process.

For 2009–23, only aggregate counts of declared substances per case were available, whereas 2024 data allowed detailed statistical analyses by substance class (demographics, associated substances, severity, and clinical effects). Descriptive analyses of sociodemographic characteristics, associated substances, severity, and reported adverse effects in 2024 were performed only for substance classes with >10 notifications. Substances were classified by chemical family, and clinical effects were coded according to the Medical Dictionary for Regulatory Activities (MedDRA) terminology at both the System Organ Class and Preferred Term levels. The severity of cases was assessed using the World Health Organization criteria for adverse drug reactions. Severe cases were defined as those resulting in death, life-threatening condition, hospitalization or its prolongation, persistent or significant disability/incapacity, or any other medically significant condition. Cases not meeting these criteria were classified as non-severe.

All abbreviations of NPS mentioned in the manuscript are summarized in Supplementary Table S1.

### OPPIDUM survey (observation des produits psychotropes illicites ou détournés de leur utilization médicamenteuse)

OPPIDUM is an annual cross-sectional survey conducted in specialized addiction treatment centers collecting data anonymously directly from users [14]. Data from 2016 to 2024 were included, focusing on the prevalence of NPS use among users and the proportion of users reporting synthetic cathinone use. Both subject-level data (number and proportion of NPS users among all participant users) and product-level data (number and proportion of NPS among all substances reported) were analyzed.

### DRAMES registry (décès en relation avec l’abus de médicaments et de substances)

DRAMES is a national mortality surveillance system collecting toxicologically confirmed deaths related to psychoactive substances [15]. Cases are identified through medico-legal investigations conducted by forensic medicine departments, which provide information on the circumstances and certified causes of death. Toxicological analyses are performed by specialized laboratories as part of the post-mortem investigation process.

All cases with identification of NPS between 2012 and 2023 (the last available data) were included. Substances were grouped into major classes (cathinones, synthetic cannabinoids, dissociatives, synthetic opioids, benzofurans, etc.) to assess temporal mortality trends.

Standardized mortality rates were calculated as the number of NPS-related deaths divided by the annual number of deaths involving psychoactive substances recorded in the program, and expressed per 100 deaths.

### Data analysis

Analyses combined descriptive and inferential statistical approaches to characterize reported patterns and trends in NPS-related notifications. Descriptive statistics were performed for each dataset. Quantitative variables (e.g. age) were summarized using mean, median, and range, whereas qualitative variables (e.g. sex, substance class, adverse effects) were expressed as absolute numbers and percentages.

Data extraction used different cut-off dates depending on the surveillance system. For the NotS database, cases with a date of creation up to 31 December 2024 were included. OPPIDUM data corresponded to the 2024 survey wave conducted in October 2024. For DRAMES, analyses included deaths occurring up to 31 December 2023. These differences reflect data availability within each surveillance system at the time of analysis.

Regression models were used to assess temporal trends. Poisson regression was initially considered; however, overdispersion was detected in the count data. Therefore, quasi-Poisson regression models with a log-link function were fitted. The logarithm of yearly totals was included as an offset to account for variations in the number of subjects across years, and year was modeled as a continuous variable. Results are presented as incidence rate ratios (IRR) with 95% confidence intervals (CI), representing the relative change in the NPS rate associated with a one-year increase.

To explore potential changes in temporal trends over time, joinpoint regression analysis was also performed using the Joinpoint Regression Program (version 5.4.0, National Cancer Institute, USA). A maximum of one joinpoint was allowed and annual percent change (APC) was estimated for each segment.

Separate models were fitted for each surveillance system (OPPIDUM, NotS and DRAMES). All statistical tests were two-sided, and statistical significance was defined as *P* < .05.

Two-sample tests for equality of proportions with continuity correction were used to compare the proportion of NPS cases observed in 2024 with those of previous years. Pairwise comparisons between 2024 and each individual year (2016–23) were performed with Bonferroni correction for multiple testing.

### Ethics

All data analyzed originated from existing national surveillance systems (ANPV, OPPIDUM, DRAMES) and were fully anonymized prior to analysis. No direct patient-identifying information was included.

## Results

### Temporal trends in addictovigilance NotS

**Figure 1.**
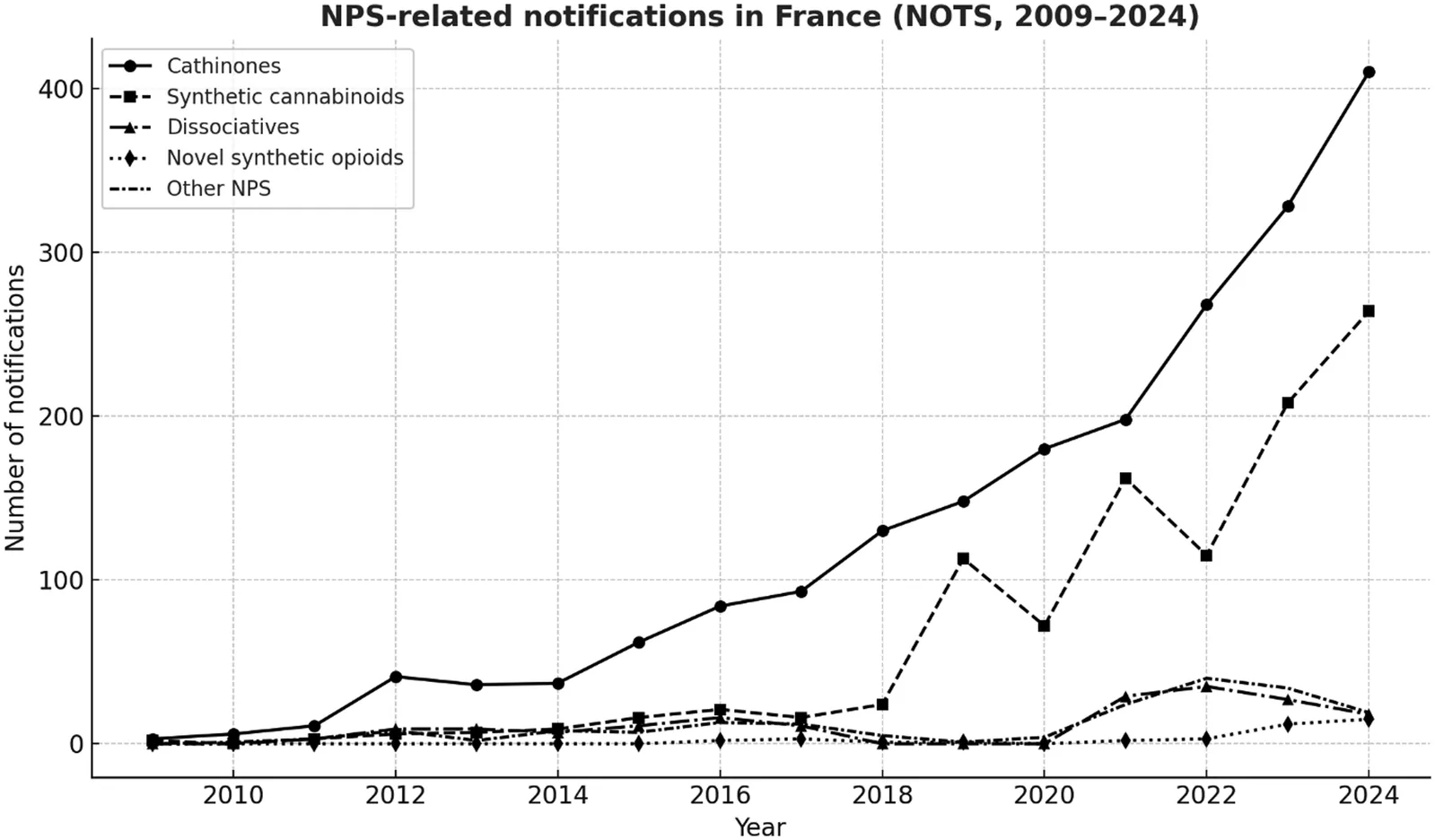
Temporal trends in new psychoactive substance (NPS)-related notifications reported to the French addictovigilance system, 2009–2024. Annual numbers of notifications involving at least one NPS are shown by substance class. The total number of notifications increased from five cases in 2009 to 726 cases in 2024. Synthetic cathinones and synthetic cannabinoids accounted for the majority of notifications throughout the study period, while other NPS classes remained marginal. The category “*Other NPS”* includes less frequently reported substances such as designer benzodiazepines, tryptamines, benzofurans, phenidates, aminoindanes, piperazines, and miscellaneous compounds (<2% each of total notifications).

As reported in Fig. 1, NPS notifications in the NotS system increased from five cases in 2009 to 726 in 2024. Quasi-Poisson regression confirmed a significant rise between 2016 and 2024 (IRR = 1.223, 95% CI, 1.16–1.28; *P* < .0001), with no change in trend identified by joinpoint analysis (annual increase: 21.7%, Supplementary Fig. S1). The proportion of NPS notifications in 2024 (10.28%) was higher than in 2016–22 (*P* < .01), but not different from 2023. Similar patterns were observed for cathinones and SC.

This increase was mainly driven by cathinones, which remained predominant, while synthetic cannabinoids rose markedly after 2019. In contrast, dissociatives declined after 2022, whereas novel synthetic opioids (NSOs), particularly nitazenes, showed a recent emergence from 2022 and stabilization in 2023–24.

**Figure 2.**
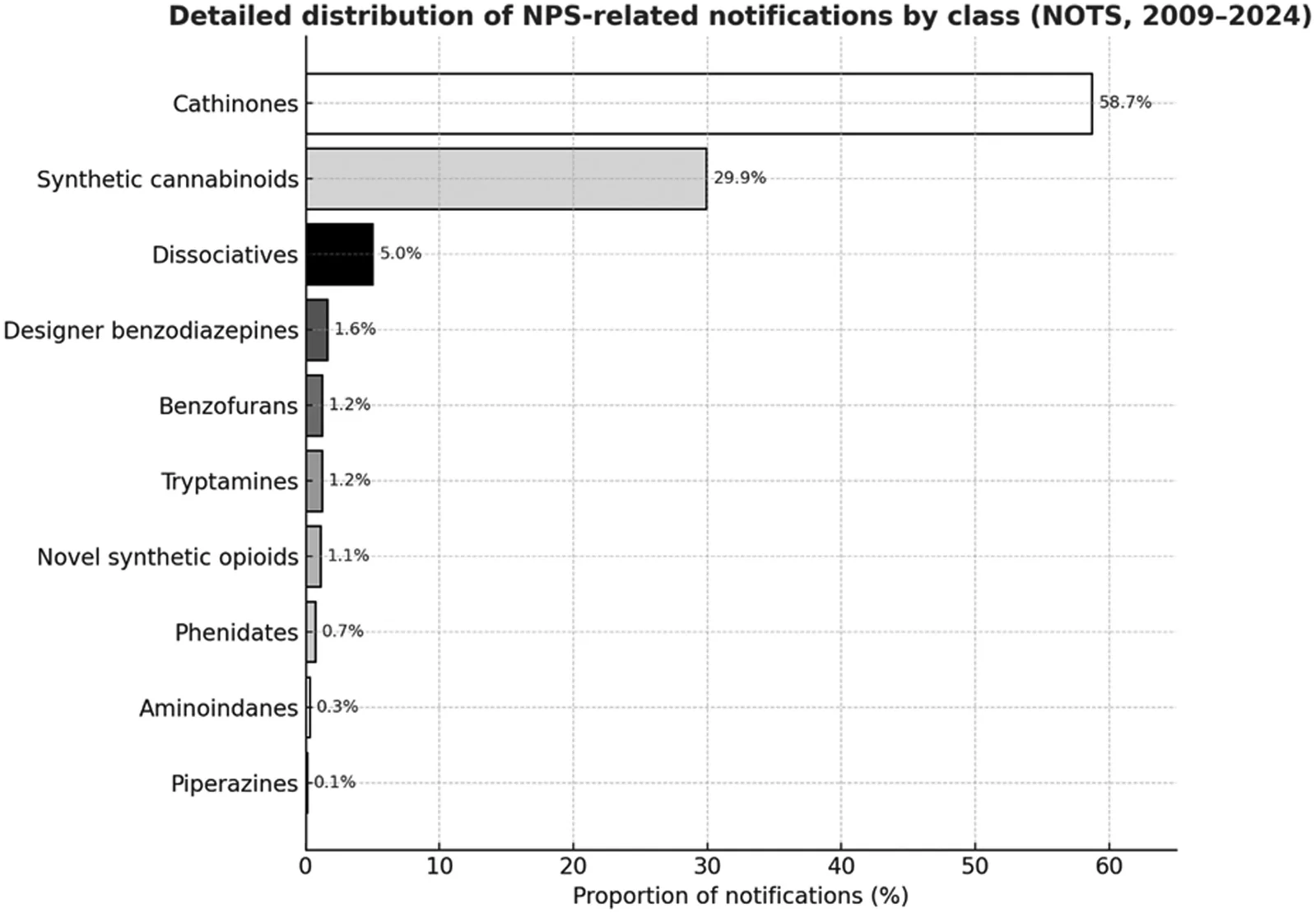
Relative distribution of new psychoactive substance (NPS) notifications reported to the French addictovigilance system, 2009–2024, by substance class. Synthetic cathinones and synthetic cannabinoids together accounted for nearly 90% of all notifications, while other NPS classes represented only a marginal proportion of reports.

As reported in Fig. 2, cathinones (58.7%) and synthetic cannabinoids (29.9%) account for nearly 90% of all notifications. Other classes [including dissociatives (5.0%), and several minor groups] represented only a marginal fraction. This distribution highlights the predominance of stimulant- and cannabinoid-type NPS, with limited but persistent circulation of other chemical families.

**Figure 3.**
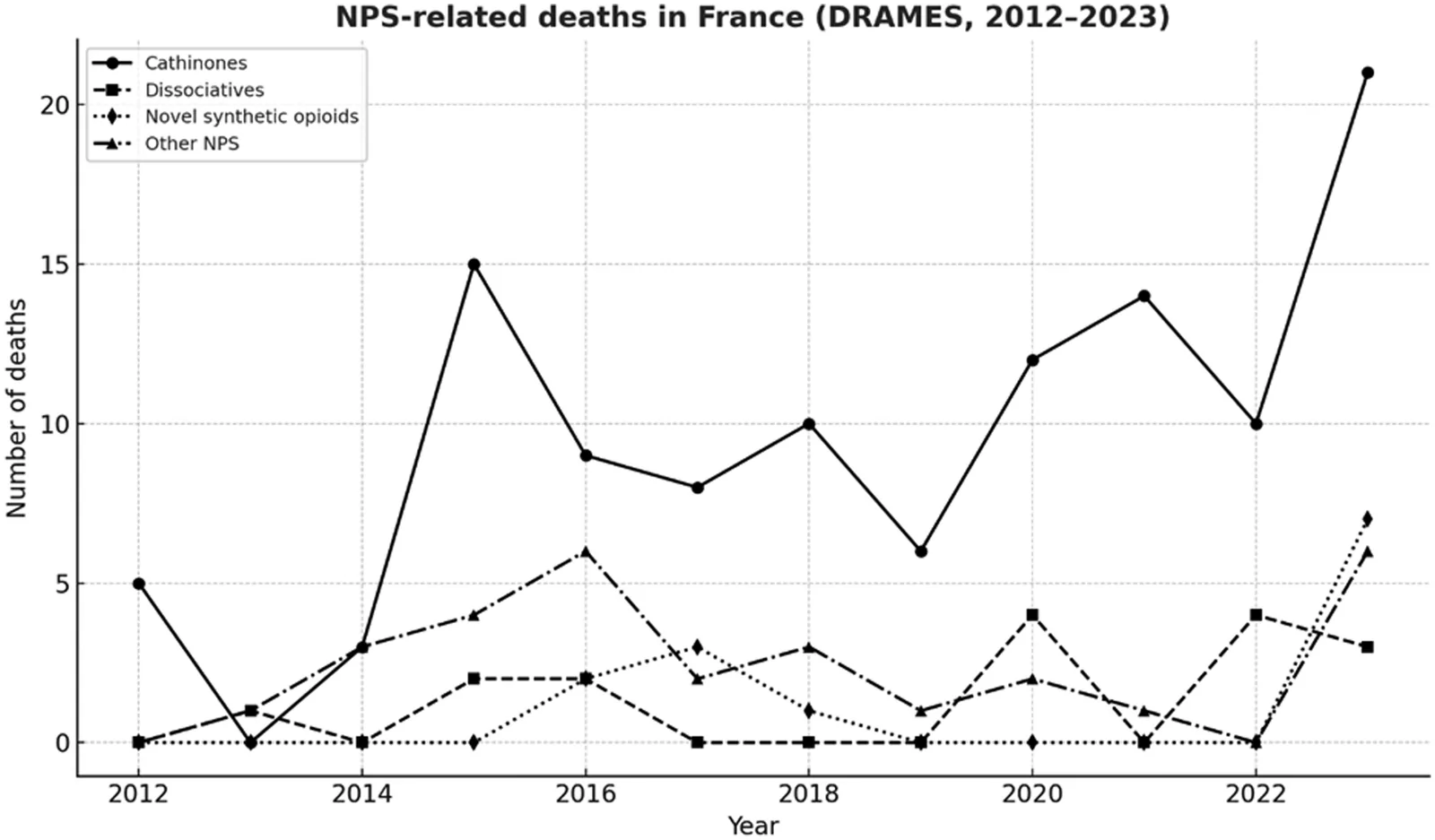
NPS-related deaths recorded in the DRAMES registry in France, 2012–2023, by substance class. The figure shows the annual distribution of NPS-related deaths by substance class. A total of 136 deaths involving at least one NPS were recorded during the study period. Distribution of NPS-related deaths reported in France between 2012 and 2023, based on DRAMES data. Synthetic cathinones accounted for the majority of NPS-related deaths over the study period, while other NPS classes contributed marginally. The category “Other NPS” includes benzofurans, designer benzodiazepines, phenidates, NBOMe derivatives, tryptamines, synthetic cannabinoids, and miscellaneous substances, each representing <5% of total deaths.

### Focus on cathinones reports (2024)

In 2024, cathinones remained the most frequently reported class of NPS, accounting for more than half of all notifications. Among the 410 addictovigilance NotS reviewed, most cases involved men (361/410, 88%). The mean age was 34 years with a median of 31 years and a range from 14 to 72 years.

A total of 456 cathinones were reported in 2024, including 125 declared as the sole substance and 331 reported in association with at least one other PS.

The detailed breakdown of cathinone subtypes highlights the predominance of X-MMC derivatives, which accounted for 75.9% of cases. Among them, 3-MMC alone represented 57.2% of all cathinone reports, confirming its central role in the French market. The second most frequent subgroup was α-pyrrolidinophenones (α-PHP, α-PVP, α-PIHP), representing 7.9% of notifications, followed by X-CMC derivatives at 5.5%. Less frequently, a limited number of cases involved other cathinones, including MDPHP and N-ethylpentedrone.

In total, 886 adverse events were reported, corresponding to a mean of 2.16 effects per notification. The most frequently reported adverse events were coma (*n* = 71), high-risk sexual behaviors (*n* = 62, reflecting chemsex practices), pharmacodependence (*n* = 49), agitation (*n* = 36), and miosis (*n* = 25).

Regarding severity, 290 cases (71%) were classified as serious, while 120 were non-serious. Fifteen deaths were reported among cathinone users in 2024.

### Focus on synthetic cannabinoids in 2024

In 2024, a total of 262 NotS involving synthetic cannabinoids were recorded. Most cases concerned men (213/262; 81.3%), with a mean age of 25 years with a median of 20 years, indicating a younger profile than that observed for cathinone cases.

A total of 315 substances were reported across these 262 cases. Among them, 166 cases involved a single SC, while 96 cases included at least one SC in combination with another PS.

The breakdown of SC subgroups highlights several distinct categories. Generic product names (e.g. Buddha Blue, PTC, “chimique”) were the most frequently reported (40.6%). The “INACA” family (e.g. BUTINACA, PINACA, CHMINACA,), accounted for 18.7% of notifications, followed by delta-9-tetrahydrocannabinol (THC) derivatives (14.6%) and hexahydrocannabinol (HHC) derivatives (12.6%). Unidentified SC (10.5%), and cannabidiol derivatives were less common (3.5%).

In total, 835 clinical effects were recorded, corresponding to a mean of 3.16 effects per notification. The most frequently reported effects were vomiting (*n* = 47), tachycardia (*n* = 36), anxiety (*n* = 30), substance abuse (*n* = 27), and somnolence (*n* = 26).

Regarding severity, 132 cases (50.4%) were classified as serious, while 130 were non-serious. One death was recorded, attributed to acute cardiac failure in the context of SC use.

### Focus on dissociatives in 2024

In 2024, a total of 18 NotS involving dissociatives (arylcyclohexylamines and diarylethylamines) were recorded. Most cases concerned men (16/18; 88.9%), with a mean age of 30.4 years and a median of 28 years (range 19–54).

A total of 19 dissociatives were declared, with 5 cases involving a single dissociative and 14 cases reported in association with at least one other PS.

The most frequently reported substances were 3-OH-PCP (*n* = 5; 27.8%), followed by 2-FDCK (*n* = 4; 22.2%), O-PCE (*n* = 3; 16.7%), and eticyclidone (*n* = 3; 16.7%). The remaining cases involved other dissociatives, including phencyclidine and related analogues.

A total of 34 clinical effects were reported, corresponding to a mean of 1.9 effects per notification. The most frequently reported effects were coma (*n* = 5), substance abuse (*n* = 4), death (*n* = 3), mydriasis (*n* = 2), and pharmacodependence (*n* = 2).

Regarding severity, 14 cases (77.8%) were classified as serious, while 4 were non-serious. Three deaths were recorded among dissociative users in 2024.

### Focus on novel synthetic opioids in 2024

In 2024, 15 NotS involving NSOs were reported. Most cases were men (13/15; 86.7%), with a mean age of 33.1 years and a median of 34 years (range 21–45).

A total of 16 NSO substances were declared, with one case involving two nitazenes (N-desethyl-protonitazene and N-desethyl-isotonitazene). In nine cases, an NSO was the sole declared substance, while six cases involved associations with at least one other PS.

The distribution of reported NSOs highlights the predominance of protonitazene (*n* = 4) and protonitazepyne (*n* = 3), followed by ketobemidone (*n* = 2), N-desethyl-protonitazene (*n* = 2), and 2-methyl-AP-237 (*n* = 2). Less frequently, carfentanil (*n* = 1) and N-desethyl-isotonitazene (*n* = 1) and metonitazene (*n* = 1) were identified.

In total, 34 adverse effects were recorded, corresponding to a mean of 2.3 effects per case. The most commonly reported adverse events were dyspnea, gait disturbances, death, and disorientation (*n* = 4 each), followed by coma (*n* = 3).

Regarding severity, 13 cases (86.7%) were classified as serious, while 2 were non-serious. Four deaths were reported, all in the context of NSO use.

A comparative summary of the main NPS classes reported in 2024 is provided in Table 1, including demographic characteristics, number of associated substances, reported adverse events, severity, and deaths. Minor classes (designer benzodiazepines, benzofurans, tryptamines, phenidates, aminoindanes) were grouped under “Other NPS.”

**Table 1.** Clinical effects associated with NPS notifications reported to the French addictovigilance system (NotS) in 2024, by major substance class^a^

Substance class	Cases, *n* (%)	Sex (M/F)	Mean age, years (median, range)	Associated substances	Adverse events, *n* (mean/case)	Severe cases, *n* (%)	Deaths, n (%)	Most frequently reported substance (%)	Main SOC categories (*n*)	Most frequent PT terms (*n*)
Synthetic cathinones	410 (56.3)	361/47	34, (31; 14–72)	125 single ; 331 ≥ 1	886 (2.16)	290 (71.0)	15 (3.7)	3-MMC (57.2)	Psychiatric (246); Nervous system (165); Socio-environmental (88)	Coma (71); High-risk sexual behavior (62); Dependence (49)
Synthetic cannabinoids	262 (36.2)	213/50	25, (20; 1–71)	166 single; 96 ≥ 1	825 (3.2)	132 (50.4)	1 (0.4)	Generic SC (Buddha Blue, PTC, “chimique”) (40.6)	Nervous system (215); Psychiatric (185); Gastrointestinal (91)	Vomiting (47); Tachycardia (36); Anxiety (30)
Dissociatives	18 (2.5)	16/2	30, (28; 19–54)	5 single; 14 ≥ 1	34 (1.89)	14 (77.8)	3 (16.7)	3-OH-PCP (27.8)	Nervous system (8); Psychiatric (7); Socio-environmental (4)	Coma (5); Substance abuse (4); Death (3);
Novel synthetic opioids	15 (2.1)	13/1	33.1 (34; 21–45)	9 single; 6 ≥ 1	34 (2.3)	13 (86.7)	4 (26.7)	Protonitazene (26.7)	General disorders/injection site (10); Psychiatric (9); Respiratory (5)	Dyspnea (4); Gait disturbance (4); Death (4);

a Clinical characteristics and severity of cases involving new psychoactive substances (NPS) are presented by substance class. Clinical effects are coded using the MedDRA terminology. “Death” corresponds to the MedDRA Preferred Term (PT) used to classify fatal outcomes reported in the notification.

Abbreviations: NPS, new psychoactive substances; PT, Preferred Term; SC, synthetic cannabinoids; SOC, System Organ Class.

### Temporal trends of OPPIDUM survey data (2016–24)

Between 2016 and 2024, 47,421 users were surveyed in the OPPIDUM program, of whom 526 (1.1%) reported using NPS. The proportion increased from 0.3% in 2016 to 1.7% in 2024.

Quasi-Poisson regression confirmed a significant increase over time (IRR = 1.175, 95% CI, 1.14–1.22; *P* = .0005). Joinpoint analysis identified a change in trend in 2018, with a sharp rise between 2016 and 2018 (APC = 62.99%, *P* < .0001), followed by a slower and non-significant increase. The proportion in 2024 remained higher than in 2016–20, with no significant difference compared with more recent years.

Detailed characteristics are presented in Supplementary Table S2.

NPS users were predominantly men (89.9%). Although the cumulative number of women reported between 2016 and 2023 was 32, 15 cases were reported in 2024 alone, suggesting an increase in the annual number of female cases. The mean age was 36.5 years (median: 36; range: 16–70). Most participants were single (73.2%) without children (90.7%). Notably, more than half reported higher education, employment, regular income, and stable housing.

Nearly three-quarters of NPS users were polysubstance consumers (median of two substances per individual, range 1–9). Routes of administration included intravenous (48.1%) and nasal use (54.2%). The main other substances reported were cocaine (30.8%), cannabis (20%), opioid substitution treatments (15.6%), and benzodiazepines (9.7%).

NPS from OPPIDUM were dominated by cathinones (81.8%), with 3-MMC alone accounting for 66.7% of all NPS cases (351 reports since 2016). Synthetic cannabinoids accounted for 5.6% of declarations, followed by hallucinogens (3.5%) and dissociatives (3.4%). In 2024, the number of cathinone reports increased slightly (*n* = 80 vs. 75 in 2023). Six new NPS were identified: α-PiHP (cathinone), B13 (cathinone), bromazolam (DBZD), deschloroetizolam (DBZD), O-PCE (arylcyclohexylamine), and THCJD (synthetic cannabinoid).

Analysis of 593 product-level reports showed that consumption was mostly weekly (42%) or occasional (38%). The main sources of supply were the internet (47%) and street dealing (40.8%).

Dependence was mentioned in 43.3% of records, abuse in 30.7%, and simple use in 22.8%. Alcohol was associated with NPS use in 27.3% of cases.

### Temporal trends of DRAMES survey data (2012–23)

In the DRAMES registry, 136 NPS-related deaths were documented in France between 2012 and 2023 (Supplementary Table S3). Between 2016 and 2023, the standardized mortality rate was 2.61 per 100 deaths involving psychoactive substances included in DRAMES (Supplementary Table S4). Quasi-Poisson regression showed no significant overall change over time (IRR = 1.02; *P* = .66). Joinpoint regression identified a change in trend in 2019, with a decrease in deaths until 2019 followed by an increase through 2023.

Cathinones were consistently the leading contributors, accounting for 82 cases (66.7%) over the study period, with deaths increasing after 2020, and peaking in 2023 (*n* = 21 cases).

Dissociatives (arylcyclohexylamines and diarylethylamines) represented the second most frequent class, with 16 deaths (13.0%), occurring sporadically across several years (2013, 2015, 2020, 2022, and 2023).

Novel synthetic opioids (NSOs) were involved in 13 deaths (10.6%), including early cases in 2016–17 and a resurgence in 2023.

Other classes accounted for isolated fatalities: benzofurans (seven cases), designer benzodiazepines (six cases), phenidates (three cases), NBOMe derivatives (two cases), tryptamines (two cases), and SC (one case in 2021). A further seven cases were classified as “other NPS.”

## Discussion

This study provides an updated overview of NPS trends in France based on three complementary addictovigilance programs (NotS, OPPIDUM, and DRAMES). Trend analyses confirmed a significant increase in NPS notifications and self-reported use in addictions care centers, indicating a diffusion of these substances in France. However, this increase was not accompanied by a significant rise in NPS-related mortality. Synthetic cathinones and synthetic cannabinoids remained predominant, accounting for nearly 90% of notifications.

Targeted investigations conducted by the French addictovigilance network facilitate early signal detection and translation into actionable information for healthcare professionals and public health authorities. These data highlight specific patterns of consumption, such as chemsex, in which cathinones are frequently involved and associated with severe psychiatric complications and high-risk sexual behaviors. The marked increase in notifications, reaching 726 cases in 2024, reflects the persistence of these substances despite regulatory controls. Market dynamics strongly influence NPS circulation, as illustrated by the substitution of 3-MMC by 3-CMC following regulatory changes in neighboring countries [16, 17]. Accumulating evidence indicates that 3-CMC may be associated with severe and sometimes fatal outcomes, underlining the need for anticipatory surveillance.

Synthetic cannabinoid notifications show the persistence of branded products alongside the emergence of chemically diverse compounds, including indazole carboxamide derivates (e.g. INACA-type compounds) and semi-synthetic cannabinoids. Clinical profiles differed markedly across classes, with cathinones mainly associated with psychiatric and neurological complications, while synthetic cannabinoids were more often linked to gastrointestinal and cardiovascular symptoms [18, 19].

OPPIDUM data confirm the penetration of cathinones among patients attending addiction care services since 2016, suggesting diffusion beyond niche user groups. However, certain populations (particularly users of synthetic cannabinoids or individuals engaged in chemsex) may remain under-represented. Compared with the overall OPPIDUM population, is generally characterized by greater social vulnerability, NPS users in our study appeared more socially integrated, with higher rates of stable housing, employment, and education [14]. DRAMES data highlight the contribution of cathinones to NPS-related deaths and the emergence of synthetic opioids such as nitazenes. However, no significant increase in mortality was observed, suggesting that the diffusion of NPS has not yet translated into a measurable rise in fatal outcomes in France. European trends highlight the need for preparedness and early response [20–25].

An additional strength of the French addictovigilance system is its contribution to practical tools such as the updated *NPS Psychoactifs* guide and mobile application (2024), which provide clinicians and users with timely information on emerging substances and associated risks, supporting rapid training and improved care [26].

Several limitations should be acknowledged. Addictovigilance relies partly on spontaneous reporting, which is subject to under-reporting and reporting bias, and toxicological confirmation is not systematic in non-fatal cases. In addition, OPPIDUM data reflect a specific care-seeking population, and DRAMES captures only toxicologically confirmed deaths. Temporal increases may also reflect improved awareness, expanded toxicological screening, and regulatory changes, and should therefore be interpreted cautiously. Despite these limitations, the combination of real-time notifications, systematic surveys and mortality surveillance provides a robust framework for monitoring emerging substances and informing public health and regulatory decisions.

## Conclusion

Over the past decade, the French NPS landscape has expanded in scope and complexity. While initially dominated by synthetic cannabinoids and cathinones, recent years have seen the emergence of potent synthetic opioids with severe health consequences. The national addictovigilance system provides a comprehensive view of NPS-related harms, from acute intoxication to mortality, and enables the rapid translation of emerging signals into clinical and public health action. Continued coordination of surveillance systems, and European collaboration remain essential to anticipate and mitigate emerging drug-related threats.

## Supplementary Material

ckag106_Supplementary_Data

## Data Availability

The data underlying this article cannot be shared publicly because they derive from national addictovigilance surveillance databases and contain sensitive health-related information. Aggregated data supporting the findings are available within the article and its supplementary material.
