# Supplementary material for: Monitoring of new psychoactive substances in France: update of addictovigilance data
Source: Eur J Public Health. 2026 Jun 16;36(4):ckag106. doi: 10.1093/eurpub/ckag106 (PMC13270969; doi:10.1093/eurpub/ckag106)
Supplement: ckag106_Supplementary_Data [file ckag106_supplementary_data.zip › ejph-2026-01-om-0045-File006.docx]

**Table S1. List of abbreviations for NPS mentioned in the manuscript**

**SC** – Synthetic Cannabinoids
**NSO** – Novel Synthetic Opioids
**NPS** – New Psychoactive Substances

**Cathinones**

- **3-MMC** – 3-Methylmethcathinone
- **3-CMC** – 3-Chloromethcathinone
- **2-MMC** – 2-Methylmethcathinone
- **α-PHP** – α-Pyrrolidinohexanophenone
- **α-PVP** – α-Pyrrolidinopentiophenone
- **α-PIHP** – α-Pyrrolidinoisohexanophenone
- **NEP** – N-Ethylpentedrone
- **MDPHP** – 3′,4′-Methylenedioxy-α-pyrrolidinohexanophenone
- **N-Ethylhexedrone** – Hexen (stimulant cathinone)
- **MDPV** – Methylenedioxypyrovalerone
- **3-MEC** – 3-Methylethcathinone
- **3-MMA** – 3-Methylmethamphetamine (sometimes classed as cathinone derivative)
- **Eutylone / Pentylone / Ethylone** – β-keto amphetamines, synthetic cathinones

**Synthetic cannabinoids**

- **INACA family** – e.g., PINACA, BINACA, CHMINACA, BUTINACA (indazole- or indole-carboxamides)
- **HHC** – Hexahydrocannabinol
- **THCJD** – Tetrahydrocannabioctyl (THC derivative)
- **CBD derivatives** – Cannabidiol analogues

**Dissociatives**

- **3-OH-PCP** – 3-Hydroxyphencyclidine
- **2-FDCK** – 2-Fluorodeschloroketamine
- **O-PCE** – 2-Oxo-PCE (eticyclidone)
- **MXE** – Methoxetamine
- **PCP** – Phencyclidine

**Novel synthetic opioids (NSO)**

- **Protonitazene** – Potent benzimidazole-derived nitazene opioid
- **Isotonitazene** – Nitazene analogue
- **Metonitazene** – Nitazene analogue
- **N-Desethyl protonitazene** – Active metabolite of protonitazene
- **N-Desethyl isotonitazene** – Active metabolite of isotonitazene
- **Protonitazepyne** – Nitazene derivative
- **2-Methyl-AP-237** – Synthetic opioid analgesic
- **Carfentanil** – Highly potent fentanyl analogue

**Designer benzodiazepines (DBZD)**

- **Bromazolam** – Triazolobenzodiazepine
- **Pyrazolam** – Triazolobenzodiazepine
- **Norflurazepam** – Benzodiazepine metabolite
- **3-Hydroxyphenazepam** – Phenazepam derivative
- **Gidazepam** – Anxiolytic benzodiazepine
- **Flubromazolam** – Triazolobenzodiazepine
- **Deschloroetizolam** – Thienotriazolodiazepine

**Benzofurans**

- **6-APB** – 6-(2-Aminopropyl)benzofuran
- **5-MAPB** – 5-(2-Methylaminopropyl)benzofuran

**Other stimulants / hallucinogens**

- **2-FMA** – 2-Fluoromethamphetamine (amphetamine derivative)
- **NBOMe** – N-Benzyl oxy-methyl derivatives of substituted phenethylamines
- **5-MeO-MiPT** – 5-Methoxy-N-methyl-N-isopropyltryptamine
