# Supplementary material for: Monitoring of new psychoactive substances in France: update of addictovigilance data
Source: Eur J Public Health. 2026 Jun 16;36(4):ckag106. doi: 10.1093/eurpub/ckag106 (PMC13270969; doi:10.1093/eurpub/ckag106)
Supplement: ckag106_Supplementary_Data [file ckag106_supplementary_data.zip › ejph-2026-01-om-0045-File007.docx]

**Table S2**. Main characteristics and substance use patterns of new psychoactive substance (NPS) users participating in the OPPIDUM survey, 2016–2024.

| **Domain** | **Findings** |
| --- | --- |
| Study population | 47,421 participants; 526 NPS users (1.1%) |
| Temporal trend | Proportion of NPS users: 0.3% in 2016 (n = 17) → 1.7% in 2024 (n = 95)  Total substances reported: 97,695; NPS: 593 (0.6%)  Proportion of NPS among reported substances: 0.2% in 2016 (n = 23) → 0.9% in 2024 (n = 100) |
| Sex | Men: 473 (89.9%); Women: 47 (8.9%); sex not specified: 6 |
| Age | Mean 36.5 years; median 36 (range 16–70) |
| Socio-demographics profile | Single : 73.2%, no children : 90.7%, higher education : 50.8% ; employed : 54.0% ; regular income : 57.8% ; stable housing : 83.8% |
| Polysubstance use | 74 % polysubstance users; mean 2.7 substances (median 2; range 1–9). |
| Main associated substances | Cocaine (30.8 %), cannabis (20 %), opioid substitution treatments (15.6 %). |
| Routes of administration | Intranasal : 54.2 % ; intravenous : 48.1 %; |
| Main NPS classes (2016-2024) | Cathinones 81.8 % (3-MMC 66.7 %: of NPS); synthetic cannabinoids 5.6 %; hallucinogens 3.5 %; dissociatives 3.4 % |
| Dependence status | Dependence: 43.3 %; abuse: 30.7 %; simple use 22.8 % |
| Alcohol co-use | 27.3 % |

**Abbreviations:** NPS, new psychoactive substances; OPPIDUM, *Observation des Produits Psychotropes Illicites ou Détournés de leur Utilisation Médicamenteuse*; PS, psychoactive substance.
