# Supplementary material for: Monitoring of new psychoactive substances in France: update of addictovigilance data
Source: Eur J Public Health. 2026 Jun 16;36(4):ckag106. doi: 10.1093/eurpub/ckag106 (PMC13270969; doi:10.1093/eurpub/ckag106)
Supplement: ckag106_Supplementary_Data [file ckag106_supplementary_data.zip › ejph-2026-01-om-0045-File008.docx]

**Table S3.** Characteristics and distribution of new psychoactive substance (NPS)-related deaths reported in France (DRAMES registry, 2012–2023).

| **Domain** | **Findings** |
| --- | --- |
| Total NPS-related death | 136 deaths (including 14 indirect cases) |
| Temporal trend | Progressive increase since 2012 (n = 3), with a peak in 2023 (n = 28) |
| Sex | Men : 130 (95.6 %) ; women : 6 (4.4%) |
| Age | Mean 39.4 years, median 38 (range 19–71) |
| Main NPS class involved 2012-2023 | Cathinones: 82 deaths (66.7 %); Dissociatives: 16 deaths (11.8%); Novel synthetic opioids: 13 (7.6 %); Other NPS classes 25 (18.4%) |
| Key qualitative signals | Ten deaths occurred in a chemsex context; emergence of novel synthetic opioids and increasing involvement of 3-CMC since 2022 |

**Abbreviations:** NPS, new psychoactive substances; DRAMES, *Décès en relation avec l’abus de médicaments et de substances*; PS, psychoactive substance.
