# Supplementary material for: Monitoring of new psychoactive substances in France: update of addictovigilance data
Source: Eur J Public Health. 2026 Jun 16;36(4):ckag106. doi: 10.1093/eurpub/ckag106 (PMC13270969; doi:10.1093/eurpub/ckag106)
Supplement: ckag106_Supplementary_Data [file ckag106_supplementary_data.zip › ejph-2026-01-om-0045-File009.docx]

**Table S4. Standardized mortality rates for NPS-related deaths recorded in the DRAMES registry, 2016–2023.**

| Year | 2016 | 2017 | 2018 | 2019 | 2020 | 2021 | 2022 | 2023 | Overall |
| --- | --- | --- | --- | --- | --- | --- | --- | --- | --- |
| Standardized mortality rate  (per 100 deaths involving psychoactive substances recorded in DRAMES) | 3.44 | 2.77 | 2.37 | 0.99 | 2.65 | 2.39 | 2.19 | 3.83 | 2.61 |

Rates were calculated using as denominator the annual number of deaths involving psychoactive substances recorded in the DRAMES programme.
